# Tris(2-hydroxy­ethyl)ammonium 1,3-benzo­thia­zole-2-thiol­ate

**DOI:** 10.1107/S160053680902248X

**Published:** 2009-06-20

**Authors:** Ji-Qin Zhu, Hua-Cai Fang, Bi-Yun Chen, Mao-Song Feng, Jing-Ning Li

**Affiliations:** a School of Chemistry and the Environment, South China Normal University, Guangzhou 510631, People’s Republic of China

## Abstract

In the title compound, C_6_H_16_NO_3_^+^·C_7_H_4_NS_2_^−^, the cations and anions are connected by O—H⋯N and O—H⋯S hydrogen bonding. Weak C—H⋯O hydrogen bonding between adjacent cations helps to stabilize the crystal structure.

## Related literature

For related structures, see Bethge *et al.* (2008 ▶); Siracusa *et al.* (2008 ▶); Solar *et al.* (2008 ▶); Varlamov *et al.* (2005 ▶).
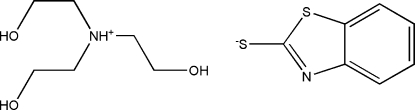


## Experimental

### 

#### Crystal data

C_6_H_16_NO_3_^+^·C_7_H_4_NS_2_^−^
*M* *_r_* = 316.43
Monoclinic, 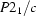

*a* = 16.496 (2) Å
*b* = 5.7184 (8) Å
*c* = 17.462 (3) Å
β = 111.524 (2)°
*V* = 1532.3 (4) Å^3^
*Z* = 4
Mo *K*α radiation
μ = 0.36 mm^−1^
*T* = 296 K
0.56 × 0.38 × 0.23 mm

#### Data collection

Bruker SMART area-detector diffractometer
Absorption correction: multi-scan (*SADABS*; Sheldrick, 1996 ▶) *T*_min_ = 0.803, *T*_max_ = 0.921
7572 measured reflections
2827 independent reflections
2185 reflections with *I* > 2σ(*I*)
*R*_int_ = 0.034

#### Refinement

*R*[*F* ^2^ > 2σ(*F* ^2^)] = 0.035
*wR*(*F* ^2^) = 0.092
*S* = 1.04
2827 reflections
185 parameters
H-atom parameters constrained
Δρ_max_ = 0.24 e Å^−3^
Δρ_min_ = −0.16 e Å^−3^

### 

Data collection: *SMART* (Bruker, 1998 ▶); cell refinement: *SAINT* (Bruker, 1999 ▶); data reduction: *SAINT*; program(s) used to solve structure: *SHELXTL* (Sheldrick, 2008 ▶); program(s) used to refine structure: *SHELXTL*; molecular graphics: *SHELXTL*; software used to prepare material for publication: *SHELXTL*.

## Supplementary Material

Crystal structure: contains datablocks I, global. DOI: 10.1107/S160053680902248X/xu2531sup1.cif

Structure factors: contains datablocks I. DOI: 10.1107/S160053680902248X/xu2531Isup2.hkl

Additional supplementary materials:  crystallographic information; 3D view; checkCIF report

## Figures and Tables

**Table 1:** Hydrogen-bond geometry (Å, °)

*D*—H⋯*A*	*D*—H	H⋯*A*	*D*⋯*A*	*D*—H⋯*A*
O1—H1⋯N1^i^	0.82	1.96	2.770 (2)	168
O2—H2⋯S2^i^	0.82	2.43	3.2258 (17)	165
O3—H3⋯S2	0.82	2.35	3.1621 (16)	169
C8—H8*A*⋯O1^ii^	0.97	2.50	3.385 (3)	151
C10—H10*B*⋯O3^iii^	0.97	2.47	3.425 (3)	167

Symmetry codes: (i) 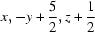; (ii) 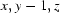; (iii) 
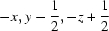
.

**Fig. 1.**
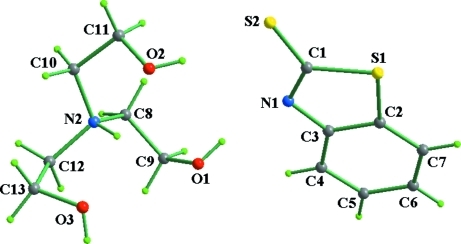
The structure of the title compound with 50% probability displacement ellipsoids.

## supplementary crystallographic information

### Comment

Some related compounds involving the 2-mercaptobenzothiazole and its derivatives has reported previously (Varlamov *et al.*, 2005; Solar *et al.*, 2008; Siracusa *et al.* 2008; Bethge *et al.*, 2008). The crystal structure of the title compound consists of tris(2-hydroxyethyl)ammonium cations and benzothiazole-2-thiolate anions (Fig. 1). The cations and anions are connected by O—H···N and O—H···S hydrogen bonding (Table 1).

### Experimental

A mixture of benzothiazole (335 mg, 2 mmol), triethanolamine (0.4 ml and 3 mmol) in ethyl acetate (20 ml) was refluxed for 20 h. The resultant yellow solution was delaminated into two layers at room temperature and then filtered. Single crystals suitable for X-ray diffraction were obtained in two day by slow diffusion of diethyl ether into a dilute solution of the title complex in ethyl acetate. The elemental analysis; calculated for C_13_H_20_N_2_O_3_S_2_: C 49.37, H 6.33, N 8.86%; found: C 49.31, H 6.38, N 8.82%.

### Refinement

H atoms were placed in idealized positions with C—H = 0.93 or 0.97 Å, O—H = 0.82 Å, N—H = 0.91 Å, and refined in riding-model approximation. *U*_iso_(H) = 1.5*U*_eq_(O) and 1.2*U*_eq_(N,*C*).

### Crystal data

**Table:**

C_6_H_16_NO_3_^+^·C_7_H_4_NS_2_^−^	*F*(000) = 672
*M**_r_* = 316.43	*D*_x_ = 1.372 Mg m^−^^3^
Monoclinic, *P*2_1_/*c*	Mo *K*α radiation, λ = 0.71073 Å
Hall symbol: -P 2ybc	Cell parameters from 236 reflections
*a* = 16.496 (2) Å	θ = 2.4–25.5°
*b* = 5.7184 (8) Å	µ = 0.36 mm^−^^1^
*c* = 17.462 (3) Å	*T* = 296 K
β = 111.524 (2)°	Block, colorless
*V* = 1532.3 (4) Å^3^	0.56 × 0.38 × 0.23 mm
*Z* = 4	

### Data collection

**Table:**

Bruker SMART area-detector diffractometer	2827 independent reflections
Radiation source: fine-focus sealed tube	2185 reflections with *I* > 2σ(*I*)
graphite	*R*_int_ = 0.034
φ and ω scans	θ_max_ = 25.5°, θ_min_ = 2.4°
Absorption correction: multi-scan (*SADABS*; Sheldrick, 1996)	*h* = −19→19
*T*_min_ = 0.803, *T*_max_ = 0.921	*k* = −6→6
7572 measured reflections	*l* = −20→21

### Refinement

**Table:**

Refinement on *F*^2^	Primary atom site location: structure-invariant direct methods
Least-squares matrix: full	Secondary atom site location: difference Fourier map
*R*[*F*^2^ > 2σ(*F*^2^)] = 0.035	Hydrogen site location: inferred from neighbouring sites
*wR*(*F*^2^) = 0.092	H-atom parameters constrained
*S* = 1.04	*w* = 1/[σ^2^(*F*_o_^2^) + (0.0394*P*)^2^ + 0.2793*P*] where *P* = (*F*_o_^2^ + 2*F*_c_^2^)/3
2827 reflections	(Δ/σ)_max_ < 0.001
185 parameters	Δρ_max_ = 0.24 e Å^−^^3^
0 restraints	Δρ_min_ = −0.16 e Å^−^^3^

### Special details

**Table:**

Geometry. All e.s.d.'s (except the e.s.d. in the dihedral angle between two l.s. planes) are estimated using the full covariance matrix. The cell e.s.d.'s are taken into account individually in the estimation of e.s.d.'s in distances, angles and torsion angles; correlations between e.s.d.'s in cell parameters are only used when they are defined by crystal symmetry. An approximate (isotropic) treatment of cell e.s.d.'s is used for estimating e.s.d.'s involving l.s. planes.
Refinement. Refinement of *F*^2^ against ALL reflections. The weighted *R*-factor *wR* and goodness of fit *S* are based on *F*^2^, conventional *R*-factors *R* are based on *F*, with *F* set to zero for negative *F*^2^. The threshold expression of *F*^2^ > σ(*F*^2^) is used only for calculating *R*-factors(gt) *etc*. and is not relevant to the choice of reflections for refinement. *R*-factors based on *F*^2^ are statistically about twice as large as those based on *F*, and *R*- factors based on ALL data will be even larger.

### Fractional atomic coordinates and isotropic or equivalent isotropic displacement parameters (Å^2^)

**Table:**

	*x*	*y*	*z*	*U*_iso_*/*U*_eq_	
C1	0.26675 (13)	1.1324 (4)	0.12815 (11)	0.0361 (5)	
C2	0.39268 (13)	0.8624 (4)	0.15930 (11)	0.0347 (5)	
C3	0.39516 (12)	1.0658 (3)	0.11586 (11)	0.0347 (5)	
C4	0.46629 (14)	1.1048 (4)	0.09259 (13)	0.0459 (5)	
H4	0.4692	1.2382	0.0632	0.055*	
C5	0.53229 (15)	0.9413 (5)	0.11398 (14)	0.0521 (6)	
H5	0.5803	0.9667	0.0992	0.062*	
C6	0.52890 (15)	0.7410 (4)	0.15671 (13)	0.0498 (6)	
H6	0.5743	0.6335	0.1699	0.060*	
C7	0.45902 (14)	0.6983 (4)	0.18000 (12)	0.0445 (5)	
H7	0.4565	0.5634	0.2088	0.053*	
C8	0.25776 (13)	0.6974 (4)	0.44442 (12)	0.0412 (5)	
H8A	0.2820	0.5472	0.4383	0.049*	
H8B	0.2541	0.7025	0.4986	0.049*	
C9	0.31648 (14)	0.8890 (4)	0.43727 (13)	0.0451 (5)	
H9A	0.3726	0.8760	0.4819	0.054*	
H9B	0.3257	0.8727	0.3858	0.054*	
C10	0.09961 (15)	0.6154 (4)	0.40603 (14)	0.0501 (6)	
H10A	0.1175	0.4593	0.4272	0.060*	
H10B	0.0458	0.6019	0.3585	0.060*	
C11	0.08354 (14)	0.7592 (4)	0.47092 (14)	0.0485 (6)	
H11A	0.0323	0.7022	0.4798	0.058*	
H11B	0.1330	0.7479	0.5224	0.058*	
C12	0.16366 (15)	0.6302 (4)	0.29797 (12)	0.0477 (6)	
H12A	0.1524	0.4633	0.2951	0.057*	
H12B	0.2190	0.6556	0.2916	0.057*	
C13	0.09228 (15)	0.7522 (5)	0.22968 (13)	0.0537 (6)	
H13A	0.0969	0.7155	0.1772	0.064*	
H13B	0.0359	0.6984	0.2282	0.064*	
N1	0.32350 (11)	1.2134 (3)	0.09787 (10)	0.0371 (4)	
N2	0.16844 (9)	0.7219 (3)	0.38008 (9)	0.0318 (4)	
H2A	0.1569	0.8778	0.3736	0.038*	
O1	0.28024 (11)	1.1097 (3)	0.44019 (9)	0.0515 (4)	
H1	0.2898	1.1449	0.4882	0.077*	
O2	0.07100 (11)	0.9936 (3)	0.44415 (9)	0.0581 (4)	
H2	0.0927	1.0808	0.4837	0.087*	
O3	0.09930 (10)	0.9968 (3)	0.24292 (9)	0.0533 (4)	
H3	0.1244	1.0551	0.2148	0.080*	
S1	0.29720 (3)	0.86166 (10)	0.17920 (3)	0.04177 (18)	
S2	0.16918 (4)	1.25406 (11)	0.11908 (4)	0.04881 (19)	

### Atomic displacement parameters (Å^2^)

**Table:**

	*U*^11^	*U*^22^	*U*^33^	*U*^12^	*U*^13^	*U*^23^
C1	0.0443 (12)	0.0320 (12)	0.0297 (10)	−0.0048 (9)	0.0107 (9)	−0.0028 (9)
C2	0.0382 (11)	0.0340 (12)	0.0270 (9)	−0.0052 (9)	0.0063 (8)	−0.0007 (9)
C3	0.0405 (11)	0.0326 (11)	0.0289 (9)	−0.0054 (9)	0.0103 (8)	−0.0021 (9)
C4	0.0496 (13)	0.0463 (15)	0.0453 (12)	−0.0018 (11)	0.0215 (10)	0.0073 (11)
C5	0.0451 (13)	0.0620 (17)	0.0543 (14)	−0.0008 (11)	0.0245 (11)	−0.0012 (12)
C6	0.0465 (13)	0.0513 (16)	0.0478 (13)	0.0115 (11)	0.0128 (11)	−0.0004 (11)
C7	0.0509 (13)	0.0388 (13)	0.0379 (11)	0.0017 (10)	0.0094 (10)	0.0031 (10)
C8	0.0392 (12)	0.0404 (13)	0.0411 (11)	0.0054 (9)	0.0114 (9)	0.0034 (10)
C9	0.0449 (12)	0.0458 (14)	0.0457 (12)	−0.0063 (10)	0.0181 (10)	−0.0091 (11)
C10	0.0506 (13)	0.0500 (15)	0.0526 (13)	−0.0124 (11)	0.0225 (11)	−0.0010 (11)
C11	0.0449 (13)	0.0574 (16)	0.0485 (13)	−0.0021 (11)	0.0234 (11)	0.0049 (12)
C12	0.0649 (15)	0.0422 (14)	0.0375 (11)	0.0001 (11)	0.0204 (11)	−0.0070 (10)
C13	0.0533 (14)	0.0671 (18)	0.0342 (11)	−0.0093 (12)	0.0082 (10)	0.0001 (11)
N1	0.0436 (10)	0.0307 (10)	0.0375 (9)	−0.0019 (8)	0.0154 (8)	0.0030 (7)
N2	0.0351 (9)	0.0275 (9)	0.0319 (8)	0.0001 (7)	0.0110 (7)	0.0011 (7)
O1	0.0690 (10)	0.0380 (10)	0.0454 (8)	−0.0041 (8)	0.0186 (8)	−0.0074 (7)
O2	0.0705 (11)	0.0570 (12)	0.0490 (9)	0.0164 (9)	0.0244 (8)	0.0038 (8)
O3	0.0591 (10)	0.0590 (12)	0.0462 (9)	0.0122 (8)	0.0244 (7)	0.0144 (8)
S1	0.0457 (3)	0.0401 (3)	0.0402 (3)	−0.0032 (2)	0.0166 (2)	0.0092 (2)
S2	0.0518 (4)	0.0470 (4)	0.0536 (4)	0.0074 (3)	0.0263 (3)	0.0074 (3)

### Geometric parameters (Å, °)

**Table:**

C1—N1	1.317 (2)	C9—H9A	0.9700
C1—S2	1.707 (2)	C9—H9B	0.9700
C1—S1	1.765 (2)	C10—N2	1.497 (2)
C2—C7	1.385 (3)	C10—C11	1.500 (3)
C2—C3	1.398 (3)	C10—H10A	0.9700
C2—S1	1.733 (2)	C10—H10B	0.9700
C3—N1	1.391 (2)	C11—O2	1.410 (3)
C3—C4	1.393 (3)	C11—H11A	0.9700
C4—C5	1.379 (3)	C11—H11B	0.9700
C4—H4	0.9300	C12—N2	1.501 (2)
C5—C6	1.379 (3)	C12—C13	1.505 (3)
C5—H5	0.9300	C12—H12A	0.9700
C6—C7	1.378 (3)	C12—H12B	0.9700
C6—H6	0.9300	C13—O3	1.415 (3)
C7—H7	0.9300	C13—H13A	0.9700
C8—N2	1.496 (2)	C13—H13B	0.9700
C8—C9	1.497 (3)	N2—H2A	0.9100
C8—H8A	0.9700	O1—H1	0.8200
C8—H8B	0.9700	O2—H2	0.8200
C9—O1	1.405 (3)	O3—H3	0.8200
			
N1—C1—S2	127.19 (16)	N2—C10—H10A	109.3
N1—C1—S1	113.51 (15)	C11—C10—H10A	109.3
S2—C1—S1	119.24 (11)	N2—C10—H10B	109.3
C7—C2—C3	121.86 (19)	C11—C10—H10B	109.3
C7—C2—S1	129.44 (16)	H10A—C10—H10B	107.9
C3—C2—S1	108.70 (15)	O2—C11—C10	108.40 (17)
N1—C3—C4	125.02 (18)	O2—C11—H11A	110.0
N1—C3—C2	115.84 (17)	C10—C11—H11A	110.0
C4—C3—C2	119.13 (19)	O2—C11—H11B	110.0
C5—C4—C3	118.6 (2)	C10—C11—H11B	110.0
C5—C4—H4	120.7	H11A—C11—H11B	108.4
C3—C4—H4	120.7	N2—C12—C13	110.28 (18)
C4—C5—C6	121.7 (2)	N2—C12—H12A	109.6
C4—C5—H5	119.2	C13—C12—H12A	109.6
C6—C5—H5	119.2	N2—C12—H12B	109.6
C7—C6—C5	120.7 (2)	C13—C12—H12B	109.6
C7—C6—H6	119.6	H12A—C12—H12B	108.1
C5—C6—H6	119.6	O3—C13—C12	109.59 (18)
C6—C7—C2	118.0 (2)	O3—C13—H13A	109.8
C6—C7—H7	121.0	C12—C13—H13A	109.8
C2—C7—H7	121.0	O3—C13—H13B	109.8
N2—C8—C9	110.98 (17)	C12—C13—H13B	109.8
N2—C8—H8A	109.4	H13A—C13—H13B	108.2
C9—C8—H8A	109.4	C1—N1—C3	111.49 (17)
N2—C8—H8B	109.4	C8—N2—C10	112.46 (15)
C9—C8—H8B	109.4	C8—N2—C12	112.13 (15)
H8A—C8—H8B	108.0	C10—N2—C12	111.54 (16)
O1—C9—C8	110.92 (17)	C8—N2—H2A	106.8
O1—C9—H9A	109.5	C10—N2—H2A	106.8
C8—C9—H9A	109.5	C12—N2—H2A	106.8
O1—C9—H9B	109.5	C9—O1—H1	109.5
C8—C9—H9B	109.5	C11—O2—H2	109.5
H9A—C9—H9B	108.0	C13—O3—H3	109.5
N2—C10—C11	111.68 (18)	C2—S1—C1	90.44 (9)
			
C7—C2—C3—N1	178.88 (18)	S2—C1—N1—C3	−178.71 (15)
S1—C2—C3—N1	−1.0 (2)	S1—C1—N1—C3	−1.5 (2)
C7—C2—C3—C4	0.1 (3)	C4—C3—N1—C1	−179.62 (19)
S1—C2—C3—C4	−179.79 (15)	C2—C3—N1—C1	1.7 (2)
N1—C3—C4—C5	−179.2 (2)	C9—C8—N2—C10	−153.34 (17)
C2—C3—C4—C5	−0.5 (3)	C9—C8—N2—C12	80.0 (2)
C3—C4—C5—C6	0.7 (3)	C11—C10—N2—C8	72.5 (2)
C4—C5—C6—C7	−0.4 (3)	C11—C10—N2—C12	−160.57 (18)
C5—C6—C7—C2	−0.1 (3)	C13—C12—N2—C8	−153.34 (18)
C3—C2—C7—C6	0.2 (3)	C13—C12—N2—C10	79.5 (2)
S1—C2—C7—C6	−179.94 (16)	C7—C2—S1—C1	−179.76 (19)
N2—C8—C9—O1	55.1 (2)	C3—C2—S1—C1	0.09 (14)
N2—C10—C11—O2	49.9 (2)	N1—C1—S1—C2	0.85 (15)
N2—C12—C13—O3	48.2 (2)	S2—C1—S1—C2	178.27 (12)

### Hydrogen-bond geometry (Å, °)

**Table:**

*D*—H···*A*	*D*—H	H···*A*	*D*···*A*	*D*—H···*A*
O1—H1···N1^i^	0.82	1.96	2.770 (2)	168
O2—H2···S2^i^	0.82	2.43	3.2258 (17)	165
O3—H3···S2	0.82	2.35	3.1621 (16)	169
C8—H8A···O1^ii^	0.97	2.50	3.385 (3)	151
C10—H10B···O3^iii^	0.97	2.47	3.425 (3)	167

Symmetry codes: (i) *x*, −*y*+5/2, *z*+1/2; (ii) *x*, *y*−1, *z*; (iii) −*x*, *y*−1/2, −*z*+1/2.
